# Editorial: Evidence-Based Public Health: Why, What and How

**DOI:** 10.3389/fpubh.2017.00253

**Published:** 2017-09-22

**Authors:** Daniel Vujcich

**Affiliations:** ^1^School of Medicine, University of Notre Dame Australia, Fremantle, WA, Australia

**Keywords:** evidence-based medicine, public health, policy, methods, theory

**Editorial on the Research Topic**

**Evidence-Based Public Health: Why, What and How**

## Introduction

In 2009, Brownson et al. (1) noted the growing consensus that evidence-based public health (EBPH) decision-making is influenced by three factors—namely, (1) best available evidence, (2) resources, and (3) population needs, values, and preferences. While this model remains both popular and plausible, we are still some way from a fulsome understanding of how its constituent parts are (or ought to be) applied in real world decision-making contexts. The articles published in this Research Topic highlight some unanswered questions with respect to the model and offer guidance for future research and practice.

## Best Available Evidence

In their policy case study, Vujcich et al. show how Australian decision-makers predominantly relied on expert opinion, qualitative research, and “lower level” quantitative studies to design interventions to reduce Indigenous smoking rates. The use of these forms of evidence exposed decision-makers to charges of “random guesses, and presumptuous rashness” (2). However, Vujcich et al. argue that the decision to introduce Indigenous-specific tobacco control initiatives was justifiable given: (a) the difficulties of rigorously measuring the effectiveness of interventions (e.g., through randomized controlled trials) in small, hard-to-reach populations and (b) the risk that the gap between Indigenous and non-Indigenous health outcomes would persist, or widen, in the absence of an intervention.

In addition to questions of equity, Fischer and Ghelardi offer a further rationale for the different understandings of “best evidence” in public health, compared to other fields such as medicine. In medicine, it is assumed that the *status quo* should be maintained until an intervention is proven to be (at least as/more) effective, and the risk of intervention-related harm is shown to be sufficiently negligible. By contrast, public health decisions typically start with the assumption that the *status quo* ought to be altered to prevent future harms; as such, a lower standard of “proof” for effectiveness is required to justify change.

The logic of this observation is made apparent in DeNicola et al. review of Saudi Arabian road traffic policies. The review describes a number of interventions that have been introduced to prevent traffic-related injuries, many of which have not been subject to randomized controlled trials due to the ethical issues around withholding potentially life-saving interventions from a group (e.g., seatbelt laws and prohibitions on drink driving). These examples make it clear that, in many public health contexts, the harms associated with inaction outweigh the potential harms which may arise from acting in the absence of “gold standard” levels of proof.

## Resources

A corollary of accepting lower standards of proof is that a greater number of interventions are likely to be deemed effective in the context of public health. In a world of scarce resources, economic analyses are therefore important in determining which of the effective interventions ought to be prioritized over others. However, as Fischer and Ghelardi note, economic analyses are not consistently conducted for public health interventions, and researchers and practitioners must do more to ensure that the “resource” component of the EBPH model is considered.

In their perspective article, Broughton and Marquez support this observation by highlighting the dearth of economic analyses on systems-level interventions to reduce medical errors; they surmise that the lack of research may reflect the complexity of accurately measuring costs and effects beyond the level of the individual. However, the authors argue that “complexity is no excuse” and offer guidance for overcoming common challenges. The solutions described support the necessary role of subjective probabilities and informed assumptions in the decision-making process when cost or feasibility impact on data collection.

## Population Needs, Values, and Preferences

Fischer and Ghelardi point to additional challenges associated with economic analyses in the case of interventions to prevent catastrophic events (e.g., climate change). It is argued that “the greater the threat, the greater a nation’s risk aversion, so the looser the cost-effectiveness threshold becomes.” While Fischer and Ghelardi do not address questions of values and ethics in detail, it is clear that such “non-economic” concepts play some role in the process. For instance, whether the nation will act to mitigate climate change depends, in part, on the nation’s perception of its responsibility to future generations.

The Indigenous tobacco control case study further highlights the importance of population values and preferences in the decision-making process. Policymakers spoke of a desire to favor proposals advanced by Indigenous community members to gain trust and support, and to guard against perceptions of paternalism. However, the case study raises important questions about how a population’s values and preferences are determined, and who has the authority to speak on behalf of a collective. While “best evidence” can be collected from published literature and efforts can be made to quantify resources, values and preferences are far more intangible.

## Conclusion

The contributions to this research topic demonstrate that the three-factor model remains a useful heuristic device for understanding EBPH. While the debate about whether EBPH should strictly adhere to traditional hierarchies of evidence has largely been settled, more work is required to understand the role of resources and values/preferences in the decision-making process. Specifically, there is a need for greater use of economic analyses, and more methodological guidance to overcome the complexities associated with measuring costs and effects at the population level. In addition to the philosophical contributions that have been published on the subject of public health ethics, more empirical and methodological research is also needed to ascertain how values and preferences are (and should be) identified and incorporated into decision-making processes. Put simply, there is more to EBPH than evidence, and more research is needed to develop a nuanced understanding of how all three factors interact to improve health outcomes.

## Author Contributions

The author confirms being the sole contributor of this work and approved it for publication.

## Conflict of Interest Statement

The author declares that the research was conducted in the absence of any commercial or financial relationships that could be construed as a potential conflict of interest.
